# The Effectiveness of Eye Movement Desensitization and Reprocessing Integrative Group Protocol with Adolescent Survivors of the Central Italy Earthquake

**DOI:** 10.3389/fpsyg.2017.01826

**Published:** 2017-10-23

**Authors:** Giada Maslovaric, Maria Zaccagnino, Clarice Mezzaluna, Sava Perilli, Denis Trivellato, Vittorio Longo, Cristina Civilotti

**Affiliations:** ^1^Centro di Ricerca e Studi in Psicotraumatologia, Bovisio-Masciago, Italy; ^2^Eye Movement Desensitization and Reprocessing Center for Eating Disorders, Milan, Italy; ^3^Facoltà di Scienze della Comunicazione, Istituto di Comunicazione Pubblica, University of Lugano, Lugano, Switzerland; ^4^Studi Cognitivi, San Benedetto del Tronto, Italy; ^5^Istituto Universitario Salesiano, Turin, Italy; ^6^Department of Psychology, University of Turin, Turin, Italy

**Keywords:** earthquake, EMDR, PTSD, disaster response, adolescents

## Abstract

Earthquakes, which can cause widespread territorial and socio-economic destruction, are life-threatening, unexpected, unpredictable, and uncontrollable events caused by the shaking of the surface of the earth. The psychological consequences, such as PTSD, anxiety, depression, and suicidal ideation, are well-known to clinicians and researchers. This study was conducted with the aim of evaluating the use of the Eye Movement Desensitization and Reprocessing (EMDR) Integrative Group Treatment Protocol on a sample of adolescents, after the earthquake in Central Italy on 24 August 2016. The objective of the EMDR intervention was to reduce PTSD symptoms. Before and after EMDR, specific assessment to find changes in PTSD symptoms was made using the Impact of Event Scale-Revised and through the analyses of the Subjective Units of Disturbance. The EMDR treatment was given in three sessions (T1, T2, and T3), each lasting 90 min, and the results at follow-up phase (T4) were also monitored. The results are very encouraging, showing significantly reduced PTSD symptoms in the majority of the subjects. The clinical implications and limitations will be discussed.

## Background

Earthquakes have always characterized human history as they are among the most common and devastating natural disasters. Today, despite scientific progress in increasing the predictability of seismic phenomena, earthquakes continue to cause devastating damage, and major destruction all over the world.

The consequences of earthquakes are not limited to the dangerousness of physical damage, indeed their traumatic repercussions have always been a subject of study in psychology. Post-traumatic stress disorder (PTSD) is the most-studied psychopathology resulting from earthquakes and natural disasters, due to the very high correlation ratios between earthquakes and this psychopathology, as documented in various studies (e.g., Pynoos et al., 1993; Bödvarsdóttir and Elklit, 2004). In recent years, among the various treatments and therapies for PTSD within emergency situation, various studies have indicated that EMDR (Eye Movement Desensitization and Reprocessing) therapy is particularly suitable for treating PTSD thanks to its applicability in emergency situations and its rapidity in achieving appreciable and lasting results (Konuk et al., 2006; Fernandez, 2007). EMDR is a structured psychotherapeutic method widely used to treat various psychopathologies and problems relating to traumatic events and emotionally stressful experiences, and adopts as a theoretical base the AIP model (Adaptive Information Processing), which works on insufficiently worked-through memories.

The project came into being following the intervention by the *Associazione EMDR Italia* between September and October 2016 when the receivers were students of the *Istituto di Istruzione Superiore di Amandola* (Fermo Province) who had survived the earthquake, and the aim was to treat PTSD through administration of the EMDR-IGTP (Integrative Group Treatment Protocol).

## The Seismic Events of 2016 in Central Italy

Due to its particular geodynamic position where the African and Eurasian plates converge, Italy has frequently been subjected to very strong seismic events sadly noted for the great damage they cause, above all in the zones of the center and south affected by the tectonics of the Apennines. One of the most recent seismic event in Italy, defined “Amatrice-Norcia-Visso seismic sequence” by the National Institute of Geophysics and Vulcanology, made itself felt from the end of summer 2016 to January 2017 with various tremors of magnitudes between 5.5 and 6.5 on the Richter scale.

On 24 August 2016, an earthquake with a magnitude of 6.0, with the epicenter along the Valle del Tronto between the communes of Accumoli (Rieti Province) and Arquata del Tronto (Ascoli Piceno Province) struck the regions of Abruzzo, Lazio, Marche, and Umbria in Central Italy (INGV, 2016).

The Civil Protection Department reported 299 dead, numerous injured, and serious damage throughout the area (Ricci Bitti, 2016).

Two months later, on 26 October 2016, two more tremors, with the epicenter on the Umbria-Marche boundary and magnitudes of 5.4 and 5.9, were recorded in the Macerata province communes of Castelsantangelo sul Nera and Ussita, respectively, and followed by a series of tremors with magnitudes of between 3.0 and 4.5. On 30 October 2016, a devastating 6.5-magnitude tremor, with the epicenter between the towns of Norcia, Preci, and Castelsantangelo sul Nera in the Province of Perugia, caused numerous collapses and serious damage but no victims.

On 18 January 2017, four tremors with magnitudes of 5.1, 5.5, 5.4, and 5.0 hit the previously stricken areas, with the epicenters in the Aquila Province communes of Montereale, Capitignano, and Pizzoli, and the Rieti Province commune of Cagnano Amiterno, respectively. The emergency situation was further worsened by the bad weather: an intense cold snap and heavy snowfalls with snowdrifts over a meter and a half high hampered rescue operations to the stricken populations (INGV, 2016).

### The Intervention of the Associazione EMDR Italia

After the 24 August 2016 earthquake, the *Associazione EMDR Italia* carried out a post-emergency intervention in the commune of Amandola (Fermo Province) to provide the population with specialist psychological support through a team specialized in psychotraumatology in emergency situations. The intervention started officially on 13 September with an informative meeting in the Council Room of the Amandola commune, and the various sessions were held mainly in the communal library, although specialist interventions were also held in private homes. Thanks to the collaboration of the Amandola commune, group interventions according to the EMDR-IGTP protocol were carried out in local primary and secondary schools (Cronache Fermane, 2016). The intervention in the commune of Amandola was part of a wider intervention, carried out from 26 August to 17 December 2016 in support of those struck by the earthquake in the areas of Amatrice, Norcia, Val Norcina, and the Province of Perugia, by the *Associazione EMDR Italia* together with institutional representatives, the Civil Protection, the Order of Psychologists of Umbria, and heads of the area mental health service. It involved 145 psychotherapists, all certified by the recognized accrediting association in Italy (*Associazione EMDR Italia*) to practice EMDR in emergency contexts. The intervention continued via further humanitarian missions even after the new tremors in January, at the same time as the emergencies caused by the weather (EMDR Italia, 2016; Fernandez, 2017).

### The EMDR-IGTP Protocol

The EMDR Integrative Group Protocol (EMDR-IGTP, Jarero et al., 2006, in the readapted version by Maslovaric and Fernandez, 2016) was used for the intervention.

The EMDR-IGTP was developed by members of the Mexican association AMAMECRISIS (Mexican Association for Crisis Therapy), as a result of the high need for mental health services occurring as a result of the destruction of Mexico’s Pacific coastline in 1997 by Hurricane Pauline. The team of doctors had initially designed a traditional, individually applied EMDR intervention aimed only at a limited number of children, adolescents, and adults who had lost family members or become homeless. However, on the first day in the field, those in need of treatment numbered more than 200. The AMAMECRISIS team were faced with the challenge of developing a suitable methodology to give so many needing support simultaneously an efficacious and specific treatment for trauma, such as the EMDR, initially developed to be applied to one person at a time (Jarero et al., 2006).

The EMDR-IGTP protocol combines the EMDR therapy of eight standard phases (Shapiro, 1995, 2001) with a group therapy model (Jarero et al., 1999; Artigas et al., 2000) and uses a particular form of bilateral stimulation called the Butterfly Hug, which is why the IGTP protocol is also known as the Group Butterfly Hug Protocol, together with the use of drawing tasks (Maxfield, 2008). The initial hypotheses behind the development of this protocol aimed at developing a methodology which could offer greater coverage than the individual EMDR approach and more efficacious results than traditional group therapies (Jarero et al., 2008). Originally developed for use with children, the EMDR-IGTP has shown that it can be applied also to group interventions with adolescents and adults: the protocol is structured as a form of play therapy, but has been successfully applied to disaster survivors with ages ranging from 7 to over 50 (Jarero and Artigas, 2010).

The advantages of the application of this protocol, apart from its simultaneous applicability to several subjects, are connected with the non-specificity of the setting, which must no longer necessarily be “private” and thus difficult to find in emergency situations. In addition, the IGTP protocol does not ask the subjects in the group to verbalize information regarding the trauma, the therapy can be applied over several consecutive days, there are no particular tasks to carry out between sessions, and treating several subjects makes it possible to rapidly involve many sections of the affected community. A further advantage offered by application of the IGTP protocol is that the clinical specialists can be assisted by paraprofessionals, teachers, and family members, and this makes wider application of the treatment protocol possible in particular emergency situations where the availability of professionals is limited (Luber, 2013).

The protocol modified by Maslovaric and Fernandez (2016) was designed to adapt the EMDR-IGTP protocol to the context of emergency situations in Italy. It takes about 90 min and foresees three sessions of intervention. The main differences with the original EMDR-IGTP protocol lie in the phases of Installation (phase 5), Body scan (phase 6), and Reevaluation (phase 8) (for further details, refer to Maslovaric and Fernandez, 2016).

The efficacy of the EMDR-IGPT approach has been documented in the literature by pilot studies in the field (Jarero et al., 1999, 2006; Artigas et al., 2000) and various case reports (Wilson et al., 2000; Korkmazlar-Oral and Pamuk, 2002; Fernandez et al., 2004; Birnbaum, 2007; Gelbach and Davis, 2007; Errebo et al., 2008; Zaghrout-Hodali et al., 2008).

In the specific field of earthquakes, there are as yet few studies and these present some methodological limitations, despite pointing out that EMDR seems a suitable methodology also for dealing with natural calamities (Konuk et al., 2006; Farrell et al., 2011). A study in 2006 by Konuk et al. (2006) analyzed the use of EMDR techniques in an experimental situation on more than 1500 trauma victims of the 1999 earthquake in Marmara, Turkey, (which had a magnitude of 7.6 and caused over 25,000 deaths), who were diagnosed with PTSD and treated with EMDR through a field study aimed at assessing a sample of 41 participants. The study indicated that EMDR treatment carried out with an average of five 90-min sessions was enough to eliminate PTSD symptoms in 92.7% of subjects and significantly reduce them in the others. It pointed out the advantages of EMDR in the emergency context typical of earthquake-affected populations who receive treatment in tent cities, compared to other strategies such as exposure-based cognitive behavioral therapies, or the techniques of “belief-restructuring” and “stress inoculation,” strategies which are considered inappropriate and difficult to apply given the emergency situation and chaotic conditions of tent cities. Furthermore, the techniques based on exposure which center on the stressful details of the event are generally considered unsuitable for a population exposed to high levels of anxiety, suffering many bereavements and under constant threat from the risks of further tremors (Bryant and Harvey, 2000). The study underlined that for such situations the EMDR-based approach was one of the most reccomended (American Psychiatric Association [APA], 2004), also in terms of the reduced number of sessions (from three to five, for a trauma based on a specific single event) compared to other treatments commonly used in similar situations (Van Etten and Taylor, 1998; Maxfield and Hyer, 2002). Moreover, the fact that it does not ask the subjects for an excessive amount of detail in their description of the traumatic event or for particular work to be carried out between sessions, makes it the specific treatment of choice for large-scale post-traumatic earthquake situations (Konuk et al., 2006). The EMDR approach was also evaluated as efficacious in similar conditions in a 2011 study by Farrell and colleagues, after EMDR techniques had been used in a humanitarian assistance training program following the 7.6-magnitude earthquake which struck northern Pakistan in 2005, killing more than 73,000, including over 35,000 children, and injuring over 135,000 (Farrell et al., 2008, 2011).

## The Study

### Method

Given the mode of operation of the health care providers and the humanitarian aim of the intervention, it was not possible to implement a randomized, delayed treatment condition. Here it is necessary to focus attention on certain ethical concerns (such as limited research funding versus the need for an expert research team, or the importance of a prompt intervention versus a rigorous and well-planned research design) in the context of humanitarian emergencies, based on the indications of the R2HC program (Research for Health in Humanitarian Crises, O’mathúna, 2015). There are various ethical concerns to consider in each research phase, from planning the research design to applying the protocols and reviewing the results. In each phase, it is necessary to try and bear in mind the individual needs of the receivers of the intervention, of the various groups and of all the affected population, as well as those of the rescuers, researchers, and all the staff involved.

It is essential to balance costs and benefits, to continually reassess the value of the aim of the research, which must answer concrete questions about the scientific validity of the research plan which must be appropriate to the demand, and ensure that the times of research take into account the timings and needs dictated by the humanitarian interventions and the allocation of resources. Informed consent and voluntary participation, which must in no way be a prerequisite for receiving adequate treatment or humanitarian support, are of fundamental importance in each phase of the research, as are respect for participants and the implementation of instruments which are properly structured for, and adapted to, the receivers of the intervention.

This study was conducted in accordance with the Declaration of Helsinki (2001), under the approval of the research guidelines of the *Centro di Ricerca e Studi in Psicotraumatologia* (C.R.S.P.) of Bovisio Masciago (Monza and Brianza province, Italy) and Article 10 of the “National Board of Italian Psychologists Code of Ethics for the Psychologist.” Moreover, regarding the ethical issues, the study was implemented following the request for intervention by the City of Amandola and upon the approval of the ethic panel of the EMDR Italian Association (Prot. EMDR_Amatrice, 1.0, 08-09-2016).

Prior to data collection, all subjects (and, because under-age adolescents, their parents) received complete information concerning the rationale and effectiveness of EMDR and the study procedures, and gave written informed consent for their participation in the study.

### Participants

In choosing the sample, it was decided to exclude all participants who, in the view of the care providers, had in the assessment phase shown symptoms of psychosis or dissociative disorders, or presented a clear risk of harming themselves or others, but no participant fulfilled any of these conditions. All 119 students of the *Istituto di Istruzione Superiore di Amandola* (Fermo Province) agreed to take part in the study. Of the 119, 116 gave valid answers when filling out the socio-demographic form regarding age and sex. The initial sample was thus composed of 65 males (average age 16.34; std dev 1.482) and 51 females (average age 16.22; std dev 1.604) for a total of 116 subjects aged 13–20 (average 16.28; std dev 1.531).

In a clinical and preventive perspective, support with the EMDR-IGTP protocol was made available to all participants, but here analysis will be of the data of the 45 out of 104 subjects (56.7% of the whole sample) who at T1 scored more than 24 points (possible diagnosis of PTSD). Of these, 17 (16.3%) scored from 24 to 32 points (partial PTSD), 7 (6.7%) scored from 33 to 36 points (full PTSD), and 21 (20.2%) scored more than 37 points (severe PTSD).

At T1, valid answers were given to all the items on the socio-demographic form except for the one concerning previous trauma, where a single answer was missing. All the subjects said that they were at home during the earthquake, except for one who was away from home; 42 (93.3%) said they lived at home, 3 (6.7%) away from home. None had been physically injured, only one reported injured family members, and 8 out of 45 (17.8%) reported damage to property due to the earthquake. 13 (28.9%) reported previous therapeutic treatment; 11 (25%), previous exposure to traumatic events (**Table 1**).

**Table 1:** Results of socio-demographic form, subjects with post-traumatic stress disorder (PTSD) at T1 (*N* = 45).

	*N*	%
Sex		
Male	19	42.2
Female	26	57.8
Location during earthquake		
At home	44	97.8
Away from home	1	2.2
Current habitation		
At home	42	93.3
Away from home	3	6.7
Physical injuries reported		
No	45	100.0
Yes	–	–
Family members injured		
No	44	97.8
Yes	1	2.2
Damage to property		
No	37	82.2
Yes	8	17.8
Previous therapeutic treatment		
No	32	71.1
Yes	13	28.9
Previous trauma		
No	33	75.0
Yes	11	25.0


### Procedure and Instruments

In the first treatment session (T1), a socio-demographic form was administered to collect data on sex, year at school, current living status, location during the earthquake, injuries received during the earthquake, injured family members, damage to property, previous therapeutic treatment, and previous exposure to potential traumatic events.

In the first and last treatment sessions (T1 and T3) and in the follow-up (T4), the adult version of the self-report IES-R (Impact of Event Scale Revised) questionnaire was administered, in order to assess PTSD (Weiss, 2007).

The IES-R, the updated version of the IES questionnaire (Horowitz et al., 1979), assesses the subjective distress perceived in relation to a potentially traumatic event. Each item is assessed according to a scale from 0 to 4 points, where 0 represents absence of relevance to the item and 4 extreme relevance. Of the 22 items assessed, eight relate to the Intrusion scale (items 1, 2, 3, 6, 9, 14, 16, 19, and 20), eight to the Avoidance scale (items 5, 7, 8, 11, 12, 13, 17, and 22), and six to the Hyperarousal scale (items 4, 10, 15, 18, 19, and 21). The reference scales are based on the PTSD symptoms as classified in the relative symptomatic clusters in the DSM-IV. The total score on the scale can range from a minimum of 0 to a maximum of 88 points, with a score over 24 considered indicative of possible PTSD. A score from 24 to 32 indicates a situation of “clinical concern” for PTSD and a possible diagnosis of partial PTSD, or in any case the presence of certain symptoms. A score from 33 to 36 represents the cutoff for a probable diagnosis of full PTSD, while a score over 37 indicates a possible diagnosis of severe PTSD (EMDRHAP, 2014).

For the data analysis, questionnaires with at most two omitted answers were considered valid. When one or two answers were missing, a substitute value (the average of the column) was inserted. At T1, 104 questionnaires were considered valid, of which 9 had one missing item and only 1 had two missing items.

The relative scores on the Subjective Units of Disturbance (SUD) scale at T1 and T3 were also taken into consideration. At each EMDR-IGTP session, the subjects were asked to make a drawing connected to the earthquake, to assign a score from 0 to 10 to represent the negative emotions associated with the drawing (SUD score) and to carry out bilateral stimulation four times. EMDR-IGTP treatment aims to reduce the SUD score associated with negative emotions regarding the event from the first drawing in a session to the last, and from the first session to the last. In the EMDR protocol, the reduction of the SUD score acts as an indicator for what is represented in the mind of the subject and for the negative emotions which the drawing arouses in the subject.

The results of the IES-R questionnaire of the 45 subjects with scores over 24 at T1 were monitored up to the third EMDR-IGTP administration (T3), where 36 questionnaires were considered valid, and at the follow-up (T4), where 35 were considered valid.

Analysis of the PTSD level of subjects at T1 showed 17 with partial PTSD (37.8%), 7 with full PTSD (15.6%), and 21 with severe PTSD (46.7%). The IES-R questionnaire scores went from a minimum of 24 to a maximum of 65 (average: 38.27 and std dev: 11.42).

During assessment of the follow-up at T4, the IES-R questionnaire showed 13 subjects without PTSD (37.1%), 8 with partial PTSD (22.9%), 3 with full PTSD (8.6%), and 11 with severe PTSD (31.4%), as well as 10 missing cases.

**FIGURE 1:**
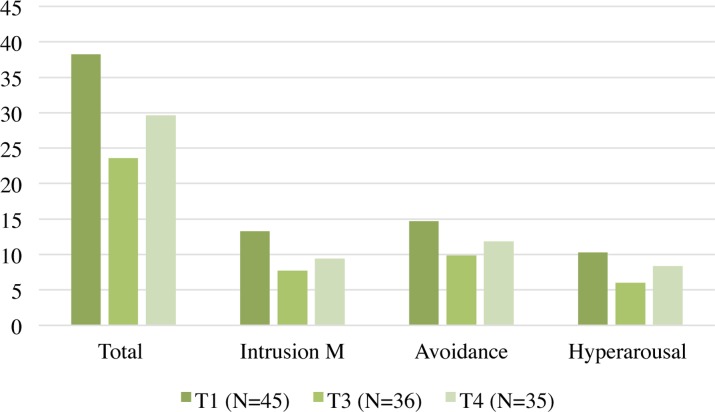
Impact of Event Scale-Revised (IES-R) scores.

The results of the total scores and of the IES-R subscales are shown in **Table 2** and **Figure 1**.

**Table 2:** Impact of Event Scale-Revised (IES-R) scores.

*T*	Total		Intrusion		Avoidance		Hyperarousal	
	*M*	*SD*	*M*	*SD*	*M*	*SD*	*M*	*SD*
T1 (*N* = 45)	38.27	11.42	13.28	6.23	14.71	3.92	10.29	4.08
T3 (*N* = 36)	23.59^∗^	12.57	7.69^∗^	4.8	9.89^∗^	5.3	6.01^∗^	4.26
T4 (*N* = 35)	29.66^∗^	15.82	9.43^∗^	6.18	11.86^∗^	6.32	8.37	5.15


^∗^, *Significant statistical difference between the averages, with p < 0.01*.

To compare the results of the IES-R and subscales at T1, T3, and T4, an ANOVA for repeated measures and a *post hoc* Bonferroni-corrected analysis were performed to determine the significance and direction of the differences of the IES-R scores relating to the first and third administrations (T1 and T3) and the measures performed at follow-up (T4).

The analysis of the total scores on the IES-R scale with *F*(2.58) = 17.195, *p* < 0.001, η^2^ = 0.37 showed statistically significant differences between T1 and T3, and between T1 and T4, but not between T3 and T4.

Analysis of the subscales showed a significant statistical difference only between T1 and T3 for the hyperarousal subscale with *F*(2.58) = 10.802, *p* < 0.001, η^2^ = 0.27; a significant difference between T1 and T3 and between T1 and T4, but not between T3 and T4 for the avoidance subscale with *F*(2.58) = 12.961, *p* < 0.001, η^2^ = 0.31; and the same for the intrusion subscale with *F*(2.58) = 14.648, *p* < 0.001, η^2^ = 0.34 (**Table 3**).

**Table 3:** Impact of Event Scale-Revised score comparisons.

	(I) IES-R total	(J) IES-R total	Mean difference (I-J)	Std. error	Sig. b	95% confidence interval for difference	
						Lower bound	Upper bound
Total score	T1	T3	16.05^∗^	2.88	0.00	8.72	23.38
		T4	9.99^∗^	2.63	0.00	3.32	16.67
Avoidance	T1	T3	5.03^∗^	0.95	0.00	2.62	7.44
		T4	3.53^∗^	1.04	0.00	0.88	6.18
Intrusion	T1	T3	6.64^∗^	1.30	0.00	3.34	9.95
		T4	4.43^∗^	1.31	0.00	1.10	7.77
Hyperarousal	T1	T3	6.64^∗^	1.30	0.00	3.34	9.95
		T4	4.43^∗^	1.31	0.00	1.10	7.77


*Comparison of pairs, correction for multiple comparison: Bonferroni, ^∗^p < 0.05*.

### Results SUD Scores

To analyze the scores on the SUD scale, an ANOVA for repeated measures and a *t*-test for paired samples were performed to verify the reduction of the SUD score at the ends of the first (SUD A, SUD B, SUD C, and SUD D at T1) and third sessions (SUD A, SUD B, SUD C, and SUD D at T3), as well as of the first and the last scores on the SUD scale from the first session to the last (SUD A and SUD D at T1 and T3).

The results of the analysis showed a significant reduction of the SUD score during the first administration between the first score (SUD A) and the third and fourth scores (SUD C and SUD D) (**Table 4**).

**Table 4:** Subjective Units of Disturbance (SUD) scores at T1 and T3.

T		Total	
		*M*	*SD*
T1 (*N* = 40)	SUD A^∗^	6.93	2.06
	SUD B	6.09	2.36
	SUD C^∗^	5.55	2.76
	SUD D^∗^	4.93	3.11
T3 (*N* = 30)	SUD A^∗^	2.93	2.377
	SUD B	2.63	2.428
	SUD C^∗^	2.27	2.149
	SUD D^∗^	1.43	1.357


^∗^*p < 0.05*.

**FIGURE 2:**
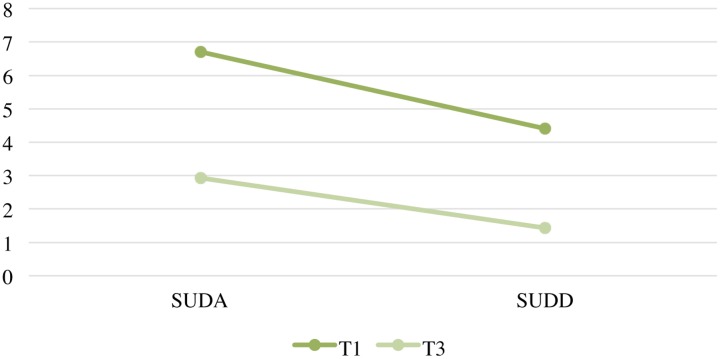
Graphic Subjective Units of Disturbance (SUD) A and SUD D at T1 and T3.

As well as the average decrease recorded in each phase of the administration, it is interesting to note, as evidence of the progressive working-through of the trauma, that also the initial levels of SUD (A) progressively diminish over time, in the same way that there is a significant reduction of the SUD linked to the final reading (D) between T1 and T3 (*p* < 0.05) (**Figure 2**).

### Discussion

The analysis of the scores reported on the IES-R and SUD scales by subjects who in the first administration had scored a total over 24 on the IES-R scale (possible diagnosis of PTSD) made it possible to hypothesize the efficacy of the EMDR-IGPT treatment in reducing in the subjects, in every phase of the intervention, both the PSTD symptoms and the negative emotiveness connected with the representations of the traumatic event.

The results of this research obtained positive confirmation with regard to the EMDR-IGTP protocol for the treatment of PTSD in a sample of adolescent survivors of an earthquake, for both the results of the IES-R scale and those of the analysis of the SUD scales.

The analysis of the IES-R scale and relative subscales makes it possible to hypothesize the efficacy of the treatment in reducing the number of subjects with probable PTSD, as seen in the comparisons between the first and final sessions of the treatment, between the first session and administration of the IES-R questionnaire in the follow-up more than 3 months later, in the total scores and in the scores on the avoidance and intrusion subscales. The analysis thus seems to confirm the efficacy of the treatment and the lasting nature of the results of application of the EMDR-IGPT protocol, as already documented in various studies and despite possible retraumatization caused by successive tremors. With regard to the hyperarousal subscale, the only significant result was the reduction between the first and last administration, and not the reduction concerning the results which emerged in the follow-up. This latter fact can be explained by the clinical significance of the hyperarousal scale, which highlights a state of alarm and continued perception of a state of possible danger. Yet considering the living conditions of the population studied (temporary housing in the stricken areas) and their exposure to a second earthquake, this does not come as a surprise. Indeed, it acknowledges the importance of a structured intervention with the dual aim of managing PTSD symptoms and preventing the worsening of the post-traumatic condition in vulnerable subjects. The fluctuating scores of the results of the IES-R questionnaire were found, although much less markedly, in other studies on the efficacy of the EMDR treatment, in particular in the reference study by Konuk et al. (2006) on the 1999 earthquake in Marmara, Turkey, which showed a substantial reduction of PTSD symptoms between the pre- and post-treatment phases, and an increase in the symptomatology, although slight, between the post-treatment and the follow-up. The differences in extent of this phenomenon between the reference study and our results may have two explanations. The first is methodological and organizational: in the study by Konuk et al. (2006), five sessions of traditional EMDR treatment were held, two more than in the EMDR-IGPT treatment applied in this research. The second concerns the continuing strong seismic activity between the various phases of the treatment of this research. While causing no victims, as there was no post-traumatic period of safety, it added to a perception of continuing danger which could both prevent consolidation in the subjects’ memory of the critical event of the first unexpected tremor and elicit negative feelings and emotions similar to those of the original event (Fernandez, 2017).

The results of analysis of the SUD scores can be used as general indicators of the therapeutic process and of the working-through of the traumatic event, in that they provide a relative indication of the negative emotional load associated with the subjects’ representations of the event (Kim et al., 2008). These results highlighted a significant reduction in the emotional disturbance of the subjects in every phase of administration, and a reduction over time of both the initial SUD levels and the final SUD scores, as evidence of the progressive working-through of the traumatic event. From a clinical point of view, because part of the IGPT protocol is to identify subjects who are not responding to the group process, it was provided additional individual EMDR work with those individuals.

### Limits of the Research

It is necessary to underline certain limits of this research determined by the humanitarian nature of the intervention, such as the relatively limited sample number, the absence of randomization procedures and the impossibility of setting up a control group, a forced choice due to the priority of guaranteeing to all receivers of the intervention treatment aimed at preventing medium- and long-term psychological disturbances arising and treatment of the acute and chronic symptoms due to post-traumatic stress.

## Conclusion

This study allows us to hypothesize the efficacy of the EMDR-IGPT intervention in a group of adolescent earthquake survivors. Today, EMDR continues to be the subject of scientific research in the field of PTSD therapy and its efficacy continues to be confirmed by many studies. However, especially in the field of emergencies, which are characterized by a series of challenges due to the event’s implicit characteristics, such as non-predictability and the ethical implications which oblige sudden intervention, there is an important difficulty in monitoring the results of the intervention.

Further studies and scientific evidence are auspicable and, as underlined by Shapiro herself, the need continues for studies concerning this issue, especially to reach a more profound understanding of the underlying mechanisms and neurobiological correlates of the treatment (Shapiro and Laliotis, 2011).

## Author Contributions

GM, MZ, and CC planned the research design and wrote the article; CM and SP contributed to the manuscript; DT and VL contributed to the statistical analyses under CC’s supervision.

## Conflict of Interest Statement

GM and MZ are offering education in EMDR field to licensed psychotherapist. GM is a coordinator of the “Emergency Section” in the EMDR Italian Association. The other authors declare that the research was conducted in the absence of any commercial or financial relationships that could be construed as a potential conflict of interest. The handling Editor declared a shared affiliation, though no other collaboration, with several of the authors, GM, SP, and DT.
